# Trench Fever Cachexia

**Published:** 1919

**Authors:** 


***Trench Fever Cachexia.*** By R. D. Rudolf. *The Lancet*, December 14, 1918.

Trench fever has now become a "recognized disease" in the army.

*Types of Trench Fever.* Two types at least have been ascertained, the short, and the long type. The short type is characterized by a continued fever lasting about 8 days, usually followed by a single short relapse. The long type commences with a short attack of fever, but is followed by a series of relapses extending over several weeks or even months. It has been proved experimentally that both types are due to the same infection.

*Cachectic Cases.* Men invalided from the front for either P. U. O. or trench fever may present a slightly abnormally high temperature, the average temperature being a degree or two higher than the normal, with occasional rises to 100° or perhaps 101°.

*Diagnosis.* In cases of this sort tuberculosis is naturally suspected, but careful investigation shows that there is nothing to confirm this suspicion.

The principal symptoms of this trench fever cachexia are chronic fever, aching shins, enlargement and hardening of the spleen. Blood examinations show nothing but a slight anemia and occasionally a slightly raised leucocyte count. Skin hyperesthesia of a peculiar distribution is frequently present in trench fever. The same distribution is found in both malaria and trench fever, and in no other disease; it corresponds to the 8th cervical, 1st and 8th dorsal, and all the lumbar segments of the cord.

Chronic trench fever has been noted to last over a year in one case, but the author supposes it may go on for much longer. Patients are usually not very ill, only in low general health with various pains. Because of its resemblance to malarial cachexia, the author suggests that this condition should be named trench fever cachexia.

